# Analytical validation of a MALDI-TOF mass spectrometry method for serum IGF-1 measurement and its comparison with LC-MS/MS and CLIA

**DOI:** 10.1016/j.plabm.2026.e00559

**Published:** 2026-09-30

**Authors:** Ruiquan Cao, Huan Ding, Yong Xia, Xueting Ma, Anping Xu, Hou-Long Luo, Ling Ji

**Affiliations:** a School of Medical Technology, Guangdong Medical University, Zhanjiang, Guangdong, China; b Department of Laboratory Medicine, Peking University Shenzhen Hospital, Shenzhen, Guangdong, China; c Intelligene Biosystems Co., Ltd., Qingdao, Shangdong, China

**Keywords:** MALDI-TOF mass spectrometry, Insulin-like growth factor 1, Analytical performance, Liquid chromatography-tandem mass spectrometry, Chemiluminescence immunoassay

## Abstract

**Background:**

Insulin-like growth factor 1 (IGF-1) is an important biomarker in the diagnosis and monitoring of growth hormone-related disorders. MALDI-TOF mass spectrometry has potential for high-throughput IGF-1 measurement, but its analytical performance for routine clinical application requires systematic validation.

**Methods:**

Analytical performance of a MALDI-TOF MS-based IGF-1 assay was evaluated following CLSI guidelines, including imprecision, accuracy, linearity, limit of quantitation (LOQ), analytical specificity, sample stability, and sample type comparison. Method comparison was performed against LC-MS/MS and CLIA using 287 patient serum samples.

**Results:**

Intra-assay coefficients of variation (CVs) were 3.66% and 1.82% at low (26.52 ng/mL) and high (375.49 ng/mL) concentrations, respectively; inter-assay CVs were 6.21% and 3.21%. The assay was linear (r = 0.998) with an LOQ of 5 ng/mL. Recoveries ranged from 87.70% to 91.73%. Cross-reactivity with IGF-2 and insulin was negligible. Serum IGF-1 remained stable for 3 days at 25°C, 9 days at 2–8°C, and 12 weeks at −20°C, and tolerated five freeze-thaw cycles. EDTA plasma and serum showed acceptable agreement (mean bias 0.36%–6.62%) for interchangeable use. Method comparison showed strong correlations: MALDI-TOF MS vs. LC-MS/MS (Passing-Bablok: Y = −5.602 + 0.746X, r = 0.996) and vs. CLIA (Y = 2.378 + 0.739X, r = 0.994).

**Conclusions:**

The MALDI-TOF MS method achieved acceptable analytical performance for serum IGF-1 measurement, including precision, linearity, stability, and specificity. The observed systematic bias relative to LC-MS/MS and CLIA suggests that method-specific reference intervals are needed. MALDI-TOF MS is a reliable and high-throughput alternative for clinical IGF-1 testing.

## Graphical abstract

**Figure:**
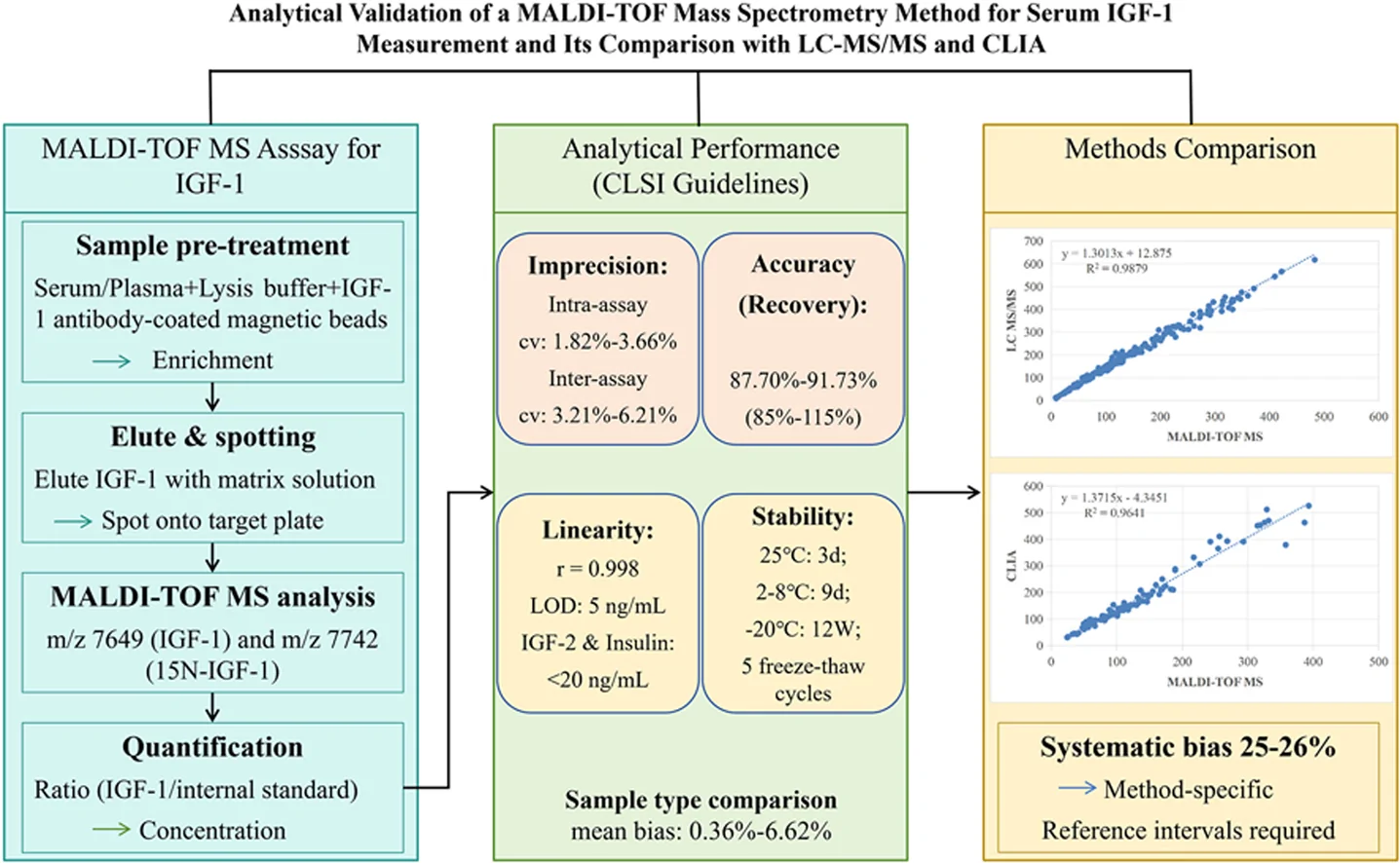

## 1 Introduction

Insulin-like growth factor 1 (IGF-1) is a 70-amino acid polypeptide hormone predominantly synthesized in the liver under the control of growth hormone (GH) [1]. In the circulation, more than 99% of IGF-1 is bound to IGF-binding proteins (IGFBPs), forming a ternary complex with IGFBP-3 and the acid-labile subunit (ALS). This complex stabilizes IGF-1 and modulates its biological availability and activity [2]. As the principal mediator of GH's somatogenic effects, including cell growth, proliferation, and differentiation, serum IGF-1 concentration reflects integrated GH secretion and is a cornerstone biomarker for GH-related disorders such as GH deficiency, gigantism, and acromegaly [[3], [4], [5], [6]]. Unlike GH, which exhibits marked pulsatility and diurnal variation, IGF-1 remains relatively stable in circulation, allowing reliable measurement from a single blood sample collected at any time of day. This stability has made IGF-1 a more practical analyte than GH for routine clinical assessment in both pediatric and adult populations [1,[6], [7], [8]].

The methodologies for IGF-1 quantification have evolved considerably over the past decades. Early radioimmunoassays and immunoradiometric assays were highly sensitive but have largely been replaced due to their complexity and radiation hazards. Enzyme-linked immunosorbent assays offered improved convenience but often lacked sufficient sensitivity and specificity. Currently, chemiluminescence immunoassay (CLIA) is the most widely used platform because of its cost-effectiveness, ease of operation, and acceptable sensitivity. However, immunoassays are subject to inter-laboratory variability, interference from endogenous binding proteins, and an inability to detect protein isoforms or variants [[9], [10], [11]]. In contrast, mass spectrometry (MS) directly measures the mass-to-charge ratio, providing high specificity, sensitivity, and the ability to detect post-translational modifications and variants. Liquid chromatography-tandem mass spectrometry (LC-MS/MS) is considered the reference method for accurate IGF-1 quantification [12,13], but its high instrumentation cost and moderate throughput have limited its adoption in routine clinical laboratories.

Matrix-assisted laser desorption/ionization time-of-flight mass spectrometry (MALDI-TOF MS) is a rapid, high-throughput platform widely used in clinical microbiology for microbial identification. Our group has previously applied MALDI-TOF MS to quantify other protein biomarkers, including hemoglobin A1c and monoclonal immunoglobulins [[14], [15], [16], [17]]. Recent studies suggest that immunoenrichment coupled with MALDI-TOF MS can achieve analytical performance comparable to LC-MS/MS for IGF-1 detection, while offering lower per-sample cost and higher throughput (over 1000 samples per day) [18]. Nevertheless, systematic validation of this approach for routine clinical application following established guidelines remains limited.

In this study, we systematically evaluated a MALDI-TOF MS-based serum IGF-1 assay following Clinical and Laboratory Standards Institute (CLSI) EP guidelines. Analytical performance parameters assessed included imprecision, accuracy, linearity, limit of quantitation (LOQ), analytical specificity, sample stability, and sample type comparability. We further compared the method against LC-MS/MS and CLIA using 287 clinical serum samples.

## 2 Materials and methods

### 2.1 Study design and ethics approval

This prospective diagnostic study was conducted in accordance with the Declaration of Helsinki (revised in 2013) and was approved by the Ethics Committee of Peking University Shenzhen Hospital. Written informed consent was obtained from all participants or their legal guardians.

### 2.2 Assays and reagents

The investigational IGF-1 assay (Intelligene Biosystems, Qingdao, China) was evaluated on the QuanTOF I automated MALDI-TOF MS platform. According to the manufacturer, the assay has a measuring range of 10–2000 ng/mL (linearity r ≥ 0.990) with a limit of detection ≤3 ng/mL. Intra-assay imprecision (CV) is ≤ 6%, and inter-assay imprecision ≤8%. Analytical specificity was defined such that quantitative detection results for 600 ng/mL IGF-2 and 18,000 μIU/mL insulin remained below 20 ng/mL.

The comparative methods were the ThermoFisher IGF-1 assay (ThermoFisher Scientific, San Jose, USA) performed on the ThermoFisher Exactive Focus LC-MS/MS platform, and the Roche Elecsys IGF-1 assay (Roche Diagnostics, Mannheim, Germany) on the Cobas e 801 CLIA platform. All assays used manufacturer-supplied calibrators and quality control materials, following standard operating procedures.

### 2.3 Study population

This cross-sectional study was conducted between November 2023 and October 2024 at Peking University Shenzhen Hospital. A total of 335 patients aged 2 to 94 years were initially enrolled. After applying exclusion criteria (insufficient sample volume, hemolysis, or incomplete demographic data), 319 patients (161 males, 50.5%) were finally included for methodological comparison and sample type analysis.

### 2.4 MALDI-TOF MS analysis

#### 2.4.1 Sample pre-treatment

Samples and quality control materials were prepared according to the manufacturer's instructions. To dissociate the IGF-1-IGFBP3-ALS ternary complex and release free IGF-1, 20 μL of lysis buffer was added to 20 μL of sample or QC material. Then, 30 μL of internal standard (^15^N-labeled IGF-1) was added, followed by IGF-1 antibody-coated magnetic beads and the corresponding buffer for IGF-1 immunoenrichment. After enrichment, the magnetic beads were washed twice with wash buffer. Subsequently, 10 μL of sinapinic acid (SA) matrix solution, prepared at 10 mg/mL in 30% acetonitrile containing 0.1% trifluoroacetic acid (TFA), was added to elute IGF-1 from the beads. Finally, 2 μL of the supernatant was spotted onto the disposable MALDI target plate. The plate was dried on an electric hot plate at 39°C for approximately 10 min and then transferred to the QuanTOF I instrument for analysis.

#### 2.4.2 Instrument parameters

The ion source voltage was set to 19 kV, with a laser frequency of 5 kHz and a laser energy of 7.0 μJ. The acquisition speed was 1 mm/s, and the mass range was set to *m*/*z* 2000–35000. Each spot was scanned with 15 laser lines. Mass spectral peak intensities for IGF-1 (*m*/*z* = 7649) and N^15^-IGF-1 (*m*/*z* = 7742) were recorded (Fig. 1A).

**Fig. 1:**
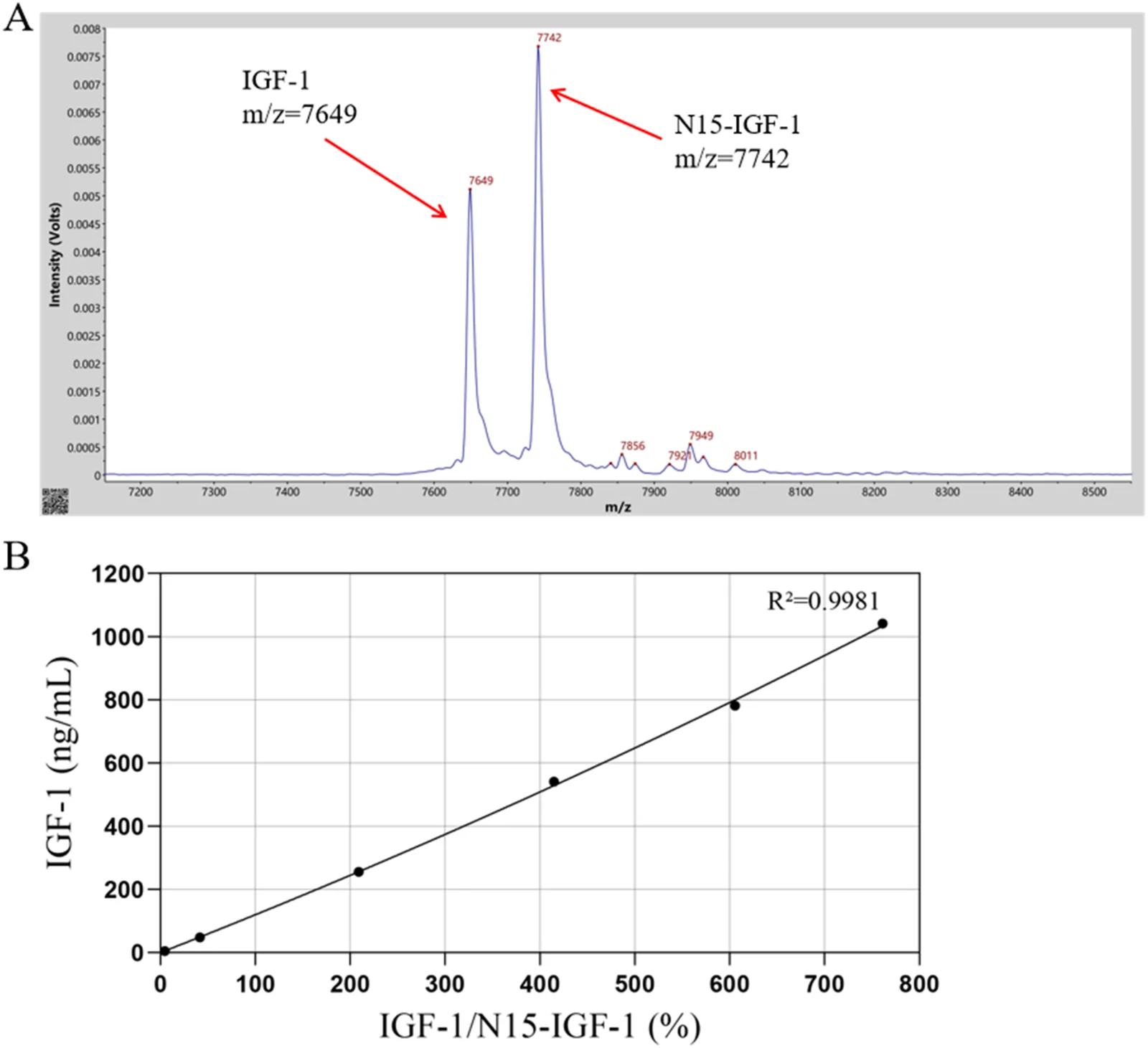
MALDI-TOF MS mass spectra of the IGF-1 calibrator. (A) Mass spectrum showing peaks corresponding to IGF-1 (*m*/*z* 7649) and N15-IGF-1 (*m*/*z* 7742). (B) Calibration curve: the ratio of IGF-1 to N15-IGF-1 measured by MALDI-TOF MS is plotted on the x-axis, and the IGF-1 concentration on the y-axis. The coefficient of determination (R^2^) for the calibration curve is 0.9981.

#### 2.4.3 Calibration curve

For calibration, 30 μL of internal standard (N^15^-IGF-1) was added to 40 μL of calibrator, followed by IGF-1 antibody-coated magnetic beads and buffer to enrich IGF-1. The subsequent steps were identical to the sample pre-treatment procedure (section 2.4.1). The calibration curve was constructed by plotting known IGF-1 concentrations (ng/mL) against the corresponding peak intensity ratios of IGF-1 to N^15^-IGF-1 obtained from the calibrators (Fig. 1B). For unknown samples, the IGF-1 concentration was determined by applying the measured IGF-1/N^15^-IGF-1 peak intensity ratio to the calibration curve.

### 2.5 LC-MS/MS analysis

Patient samples were prepared using modification of the methods previously described by Motorykin et al. [19]. Briefly, 100 μL aliquots of patient sera, QCs, and calibrators were thawed, vortexed, mixed with 10 μL ^15^N-IGF-1 internal standard and 400 μL acidified ethanol (87.5% EtOH/12.5% 1 N HCl), incubated at room temperature for 30 min, and centrifuged (5500 RCF, 10 min, 10°C). The supernatant (350 μL) was neutralized with 60 μL 1.5 M Tris base, cooled at −20°C for 60 min, and re-centrifuged under the same conditions.

IGF-1 was separated by online extraction HPLC (Aria TLX-4) using a Phenomenex Monolithic Onyx C18 Guard Cartridge for extraction and a Phenomenex Kinetex C18 analytical column (50 × 4.6 mm, 100 Å) with gradient elution of water/acetonitrile (both with 0.1% formic acid). Mass spectrometry was performed on a Q Exactive Focus with HESI source in positive AIF mode (resolution, 70,000; scan range, 1060-1110 Da; normalized collision energy (NCE), 10; automatic gain control (AGC), 2e4; maximum injection time (IT), auto) for total IGF-1 quantification, providing ∼2-fold higher sensitivity than SIM. The A67T/A70T pair was quantified by PRM (resolution 35,000; isolation window 2.0 *m*/*z*; NCE 30; fixed first mass 150 Da; AGC 2e4; IT 250 ms; 1 microscan). Data were acquired and analyzed using Thermo TraceFinder 5.0 Clinical and visualized with Thermo Xcalibur 4.2 Qual Browser.

### 2.6 CLIA analysis

The Roche Elecsys IGF-1 assay is a two-step sandwich immunoassay. IGF-1 is first released from its binding proteins by acid dissociation in the presence of blocking reagents. The liberated IGF-1 then forms a sandwich complex with two monoclonal antibodies: one conjugated to biotin and the other conjugated to the electrochemiluminescent label Ru(bpy)_3_^2+^. The complex subsequently binds to streptavidin-coated magnetic microparticles, followed by magnetic separation and washing. The electrochemiluminescent signal is measured and quantified against a calibration curve. The assay calibration is traceable to the WHO International Standard for IGF-1 (NIBSC 02/254). Commercial quality control materials (PreciControl Growth) were used for routine analytical quality control.

### 2.7 Analytical performance

All analytical performance assessments were designed and conducted in general accordance with relevant CLSI guidelines.

*Imprecision*. Imprecision was evaluated according to CLSI EP5-A3 [20]. Two serum samples at different IGF-1 concentrations were selected: a low-concentration sample (approximately 26 ng/mL) and a high-concentration sample (approximately 375 ng/mL). Each concentration level was tested in two runs per day, each run with two replicates, over 20 consecutive days. Acceptance criteria were intra-assay imprecision < 1/4 total allowable error (TEa) and inter-assay imprecision < 1/3 TEa. Based on Westgard biological variation database requirements, the TEa for IGF-1 was set at 24% [21].

*Accuracy*. Accuracy was assessed by a recovery test. A low-concentration serum sample was spiked with standard solutions of the analyte at three concentration levels to prepare recovery test samples. Each recovery sample was analyzed in triplicate, and the recovery rate was calculated as (measured concentration/expected concentration) × 100%. The acceptable recovery range was defined as 85.0% to 115.0%.

*Linearity*. Linearity was evaluated following CLSI EP6-A [22]. Eleven samples covering the full calibration range were prepared by mixing a high-concentration sample and a low-concentration sample in various proportions. Each concentration level was measured in triplicate. The dilution factor was plotted on the horizontal axis, and the corresponding mean measured value on the vertical axis. Polynomial regression analysis was applied to assess linearity.

*Limit of quantification (LOQ)*. LOQ was determined in accordance with CLSI EP17-A2 [23]. Five serum samples with concentrations near the expected lower LOQ (LLOQ) were selected. Each level was measured five times, and the mean and coefficient of variation (CV) were calculated. The lowest concentration meeting the predefined CV requirement (≤10%) was established as the validated LOQ.

*Analytical specificity*. Analytical specificity was assessed by spiking serum samples with structurally related proteins, including IGF-2 and insulin. Samples containing IGF-2 at a concentration of no less than 600 ng/mL were tested twice, and the results were required to be no higher than 20 ng/mL. Similarly, samples containing insulin at a concentration of no less than 18,000 μIU/mL were tested twice, with results also required to be no higher than 20 ng/mL.

*Sample stability*. Stability testing followed the Checklist for Reporting Stability Studies (CRESS) [24]. Fresh serum samples with high and low IGF-1 concentrations were selected and tested in triplicate; the mean value was taken as the baseline. Samples were then stored under the following conditions: ① At room temperature (25°C): tested every 24 h, three replicates per test, over three consecutive days. ② Refrigerated at 2°C to 8°C: tested every three days, three replicates per test, for 9 days. ③ Frozen at −20°C: tested every two weeks, three replicates per test, for 12 weeks. ④ Frozen at −20°C with repeated thawing: samples were thawed, tested weekly, and then refrozen; this was repeated for 5 weeks (5 freeze-thaw cycles), with three replicates per test. According to the CRESS guidelines, the percentage deviation (PD) from baseline was used to determine significant change. A PD within ±10.7% [25] was considered acceptable; otherwise, samples were regarded as unstable.

*Sample type comparison*. Comparison between paired serum and K2-EDTA plasma specimens was performed. For each of 32 patients, blood was drawn simultaneously into three tube types: tubes without additive (serum 1, plain tube), tubes with gel and clot activator (serum 2, serum separator tube), and tubes with K2-EDTA (plasma). All three sample types from the same patient were assayed in parallel, and the results were compared by Passing-Bablok regression and Bland-Altman analysis.

### 2.8 Method comparison

Following CLSI EP9-A3 guidelines [26], agreement and bias of the MALDI-TOF MS method were evaluated by comparing IGF-1 results against two reference methods: LC-MS/MS and CLIA. A total of 287 serum samples were collected from patients attending the emergency department, outpatient clinics, and inpatient wards between November 2023 and October 2024. Of these, 191 samples were analyzed in parallel by MALDI-TOF MS and LC-MS/MS. Passing-Bablok regression was performed to assess correlation, with LC-MS/MS results on the x-axis and MALDI-TOF MS results on the y-axis. The remaining 96 samples were analyzed in parallel by MALDI-TOF MS and CLIA, with Passing-Bablok regression again used (CLIA on the x-axis, MALDI-TOF MS on the y-axis). Bland-Altman analysis was applied to determine agreement and potential systematic bias between methods. Residuals exceeding 4 standard deviations (SD) of the regression were considered outliers.

### 2.9 Statistical methods

Statistical analyses were carried out using SPSS (version 27.0; IBM, Armonk, NY, USA), GraphPad Prism (version 7; GraphPad Software, San Diego, CA, USA), and MedCalc (version 23.4; MedCalc Software, Ostend, Belgium). Method comparisons, including Passing-Bablok regression and Bland-Altman analysis, were performed with MedCalc.

## 3 Results

### 3.1 Analytical performance

The MALDI-TOF MS assay showed acceptable imprecision across the tested concentration range. Intra-assay CVs were 3.66% at a low concentration (26.52 ng/mL) and 1.82% at a high concentration (375.49 ng/mL). Inter-assay CVs were 6.21% at the low concentration and 3.21% at the high concentration (Supplementary Table S1).

Linearity assessment across the calibration range yielded the regression equation Y = 1.009X – 17.564, with a correlation coefficient r = 0.998, meeting the manufacturer's claim of r ≥ 0.990 (Supplementary Fig. 1). Recoveries across the measuring range ranged from 87.70% to 91.73%, with a mean of 89.43%, all within the predefined acceptable range of 85% to 115% (Supplementary Table S2). The limit of quantitation (LOQ), defined as the lowest concentration achieving a CV of ≤10% on the imprecision profile, was determined to be 5 ng/mL (Supplementary Table S3).

Cross-reactivity was negligible for all tested potential cross-reactants. For both IGF-2 (≥600 ng/mL) and insulin (≥18,000 μIU/mL), the measured IGF-1 concentrations remained below 20 ng/mL, meeting the manufacturer's claimed interference limit (Supplementary Table S4). These results confirmed the analytical specificity of the assay.

Sample stability testing showed that serum IGF-1 was stable for up to 3 days at 25°C (percentage deviation [PD] from baseline: 1.92%–8.34%), for 9 days at 2–8°C (PD: −0.54% to 7.27%), and for 12 weeks at −20°C (PD: −4.95% to 8.52%) (Supplementary Table S5). Samples also tolerated five freeze-thaw cycles without significant degradation (PD: −2.24% to 4.29%) (Supplementary Table S5).

Sample type comparison between serum and EDTA plasma showed acceptable agreement. Passing-Bablok regression and Bland-Altman analysis gave the following results: K2-EDTA plasma versus serum 1 (plain tube): Y = 0.281 + 0.935X, r = 0.996, n = 32, mean bias 6.27%; K2-EDTA plasma versus serum 2 (serum separator tube): Y = −0.550 + 0.946X, r = 0.996, n = 32, mean bias 6.62%; serum 1 versus serum 2: Y = 1.138 + 0.984X, r = 0.996, n = 32, mean bias 0.36% (Supplementary Fig. 2). No statistically significant difference was observed between EDTA plasma and either serum type, and the biases were deemed acceptable for interchangeable use of the three specimen types.

### 3.2 Method comparison

Method comparison was performed using 191 serum samples for the MALDI-TOF MS vs. LC-MS/MS analysis and 96 serum samples for the MALDI-TOF MS vs. CLIA analysis. Each specimen was measured once.

Passing-Bablok regression revealed an excellent linear correlation between LC-MS/MS and MALDI-TOF MS across the full analytical range: Y (MALDI-TOF MS) = −5.602 + 0.746 × X (LC-MS/MS), with a correlation coefficient r = 0.996 (Fig. 2A). Bland-Altman analysis further confirmed agreement between the two methods (Fig. 2B). Residual analysis from the Passing-Bablok regression showed that no residuals exceeded 4 SD of the regression, indicating the absence of significant outliers (Fig. 2C).

**Fig. 2:**
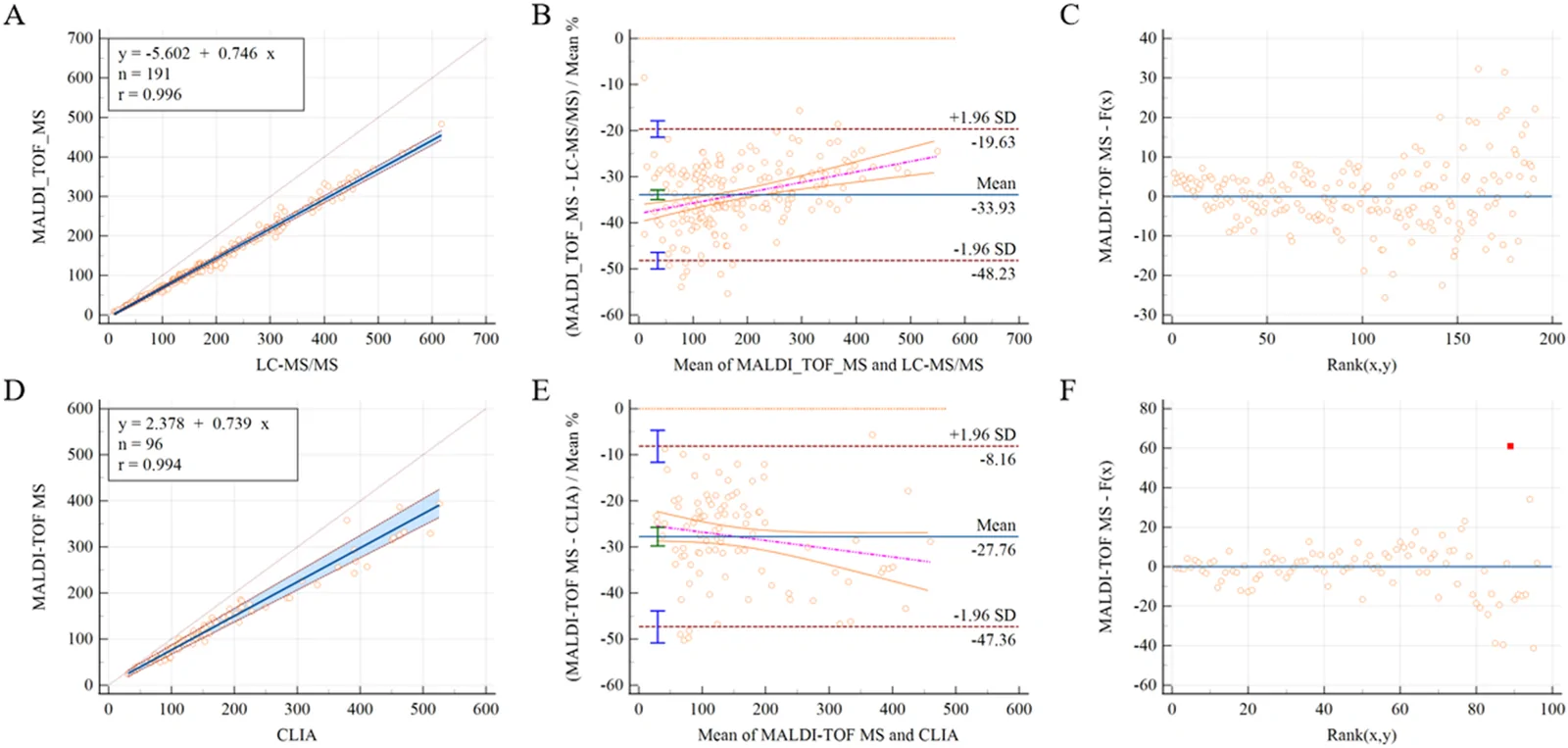
Method comparison between MALDI-TOF MS and two reference methods (LC-MS/MS and CLIA). (A) Passing-Bablok regression plot of MALDI-TOF MS versus LC-MS/MS using 191 serum samples. (B) Bland-Altman plot consistency test of MALDI-TOF MS and LC-MS/MS. (C) Residual plot from Passing-Bablok regression for MALD-TOF MS versus LC-MS/MS; no residuals exceeded 4 SD, indicating no significant outliers. (D) Passing-Bablok regression plot of MALDI-TOF MS versus CLIA using 96 serum samples. (E) Bland-Altman plot for MALDI-TOF MS versus CLIA. (F) Residual plot for MALDI-TOF MS versus CLIA; one sample (Red) showed a residual exceeding 4 SD and was identified as a significant outlier.

A strong linear relationship was also observed between CLIA and MALDI-TOF MS. Passing-Bablok regression yielded Y (MALDI-TOF MS) = 2.378 + 0.739 × X (CLIA), with r = 0.994 (Fig. 2D). Bland-Altman analysis confirmed acceptable agreement (Fig. 2E). Among the 96 samples, one sample (1.04%) was identified as a significant outlier, with a residual exceeding 4 SD (Fig. 2F). Excluding this outlier did not materially change the regression parameters (data not shown).

Taken together, these results demonstrate robust analytical concordance between the MALDI-TOF MS method and both reference methods, supporting its suitability for clinical IGF-1 measurement. However, the systematic negative bias of approximately 25–26% relative to both LC-MS/MS and CLIA indicates that method-specific reference intervals are required.

## 4 Discussion

In this study, we systematically evaluated a MALDI-TOF MS-based method for serum IGF-1 quantification following CLSI EP guidelines. The method met the predefined acceptance criteria for imprecision, accuracy, linearity, LOQ, and sample stability.

For imprecision, the intra-assay CVs were 3.66% (low concentration) and 1.82% (high concentration), and the inter-assay CVs were 6.21% and 3.21%, respectively. These values compare favorably with previously reported performance metrics for MALDI-TOF MS-based IGF-1 assays. Oran et al. described a parallel high-throughput workflow for IGF-1 quantification using mass spectrometric immunoassay, reporting a CV of approximately 9% at the lower end of the measuring range [18]. The imprecision observed in our study, particularly the 6.21% CV at the low concentration, is within clinically acceptable limits and approaches the performance of established LC-MS/MS methods. The somewhat higher variability at the low concentration likely reflects random errors introduced during manual sample preparation steps, which involved multiple manual operations including pipetting, magnetic bead enrichment, washing, and matrix application.

Recovery rates ranged from 87.70% to 91.73%, all falling within the predefined acceptable range of 85% to 115%. These values are comparable to those reported for other mass spectrometric methods. Zeng et al. reported recoveries for IGF-1 measured by high-resolution mass spectrometry generally within 80% to 120% [27]. The slight decline in recovery with increasing spiked concentrations may reflect stability differences of the calibrator material at higher concentrations. Analytical specificity was confirmed by negligible cross-reactivity with IGF-2 and insulin. Interference from these structurally related proteins remained below 20 ng/mL, confirming that the antibody used for immunoenrichment provides sufficient selectivity for IGF-1. This finding is consistent with the known high specificity of mass spectrometry-based methods compared to immunoassays, in which cross-reactivity with IGF-binding proteins and IGF-2 can produce falsely elevated or depressed results [28,29].

Stability testing demonstrated that serum IGF-1 samples were stable for 3 days at 25°C, 9 days at 2–8°C, and 12 weeks at −20°C. Samples also withstood five freeze-thaw cycles without significant degradation. These stability profiles support the preanalytical robustness of the method for routine clinical laboratory handling. A slight increase in measured values was noted in samples stored at 25°C over time; the mechanism underlying this observation warrants further investigation. Our results are consistent with the findings reported by Louw et al. [25]. Sample type comparison showed no statistically significant difference between EDTA plasma and serum, with mean biases ranging from 0.36% to 6.62% across different serum collection tubes. These findings support the interchangeable use of serum and EDTA plasma specimens for IGF-1 quantification using this MALDI-TOF MS assay. This is practically relevant because plasma collection is often preferred in emergency or high-throughput settings where rapid centrifugation and processing are required.

Method comparison against reference methods showed strong correlations between MALDI-TOF MS and both LC-MS/MS (r = 0.996) and CLIA (r = 0.994). However, a systematic negative bias of approximately 25–26% was observed relative to both comparative methods (Fig. 2B and E). This finding is not unprecedented given the known discordance between mass spectrometric and immunoassay methods for IGF-1 measurement. Zeng et al. recently reported that a chemiluminescence immunoassay showed a negative bias relative to LC-MS/MS of up to 20% [27]. Conversely, Vos et al. demonstrated that IDS iSYS IGF1 immunoassay yielded a positive proportional bias of approximately 27% relative to LC-MS/MS [30]. These observations align closely with the direction and magnitude of bias observed in our study. More recently, Yan et al. published a consistency study of MALDI-TOF MS and CLIA for IGF-1 detection in a healthy Chinese cohort of 660 individuals, reporting a strong correlation (R^2^ = 0.982) with a regression equation of IGF-1_CLIA = 0.972 × IGF-1_MALDI-TOF_MS + 5.041 and an intraclass correlation coefficient exceeding 0.80 [31]. Their Bland-Altman analysis showed a mean difference of −0.25 ng/mL, within a clinically acceptable range. The divergence between their findings and ours—specifically the minimal bias observed in their study versus the ∼25% negative bias we observed—likely reflects differences in study design, calibration traceability, and the specific MALDI-TOF MS and CLIA platforms used.

The study has several limitations. First, manual sample preparation introduced random error that likely contributed to the variability observed in imprecision studies, particularly at the lower concentration range. Implementation of fully automated sample pre-processing could help reduce such operational bias and further improve precision. Second, the establishment of method-specific reference intervals was constrained by sample availability. We were unable to collect sufficient samples covering all age groups, especially the pediatric population, which limits the generalizability of our findings. Given the age-dependent dynamics of IGF-1, comprehensive reference interval studies stratified by age and sex are essential for clinical implementation. Third, although we observed a systematic negative bias of approximately 25-26% between MALDI-TOF MS and both LC-MS/MS and CLIA, the present study did not further investigate the underlying mechanisms. Whether the bias originates from calibration differences, sample preparation protocols, or matrix effects requires clarification in future research. Finally, the sample type comparison was conducted on a relatively modest number of patients (n = 32), and although the results supported interchangeability, larger validation studies are warranted.

## 5 Conclusions

The MALDI-TOF MS method evaluated in this study showed satisfactory analytical performance for serum IGF-1 detection, meeting CLSI criteria for imprecision, linearity, accuracy, LOQ, and sample stability. The assay offers the advantages of high throughput, favorable cost-effectiveness, and the ability to detect protein variants—a capability that distinguishes mass spectrometry from immunoassay-based approaches. However, the systematic bias observed relative to established methods underscores the need for method-specific reference intervals for clinical application; universal reference intervals cannot be applied across platforms with differing analytical principles. Results obtained using this MALDI-TOF MS assay should be interpreted with caution during cross-platform comparisons or longitudinal monitoring when a patient's IGF-1 measurements have been obtained with different methods over time. Future efforts should focus on method harmonization through commutable reference materials and the establishment of robust, population-based reference intervals to facilitate the clinical adoption of this technology.

## CRediT authorship contribution statement

**Ruiquan Cao:** Writing – original draft, Methodology, Data curation, Conceptualization. **Huan Ding:** Writing – original draft, Data curation, Conceptualization. **Yong Xia:** Supervision, Resources, Formal analysis. **Xueting Ma:** Methodology, Data curation. **Anping Xu:** Resources, Methodology, Formal analysis. **Hou-Long Luo:** Writing – review & editing, Validation, Project administration, Conceptualization. **Ling Ji:** Writing – review & editing, Funding acquisition, Conceptualization.

## Ethics declaration

This study was performed in compliance with relevant laws, regulatory frameworks and guidelines where the research took place. The Ethics Committee of Peking University Shenzhen Hospital granted an exemption. The authors provided the following explanation: This prospective diagnostic study was conducted in accordance with the Declaration of Helsinki (revised in 2013) and was approved by the Ethics Committee of Peking University Shenzhen Hospital. Written informed consent was obtained from all participants or their legal guardians.

## Funding

This work was supported by Guangdong Provincial Department of Science and Technology (Grant No. 2022B1111020001) and Qingdao Science and Technology Program (Grant No. 2023TSGC0502).

## Declaration of competing interest

The authors declare that they have no known competing financial interests or personal relationships that could have appeared to influence the work reported in this paper.

## Figures and Tables

**Supplemental Fig. 1:**
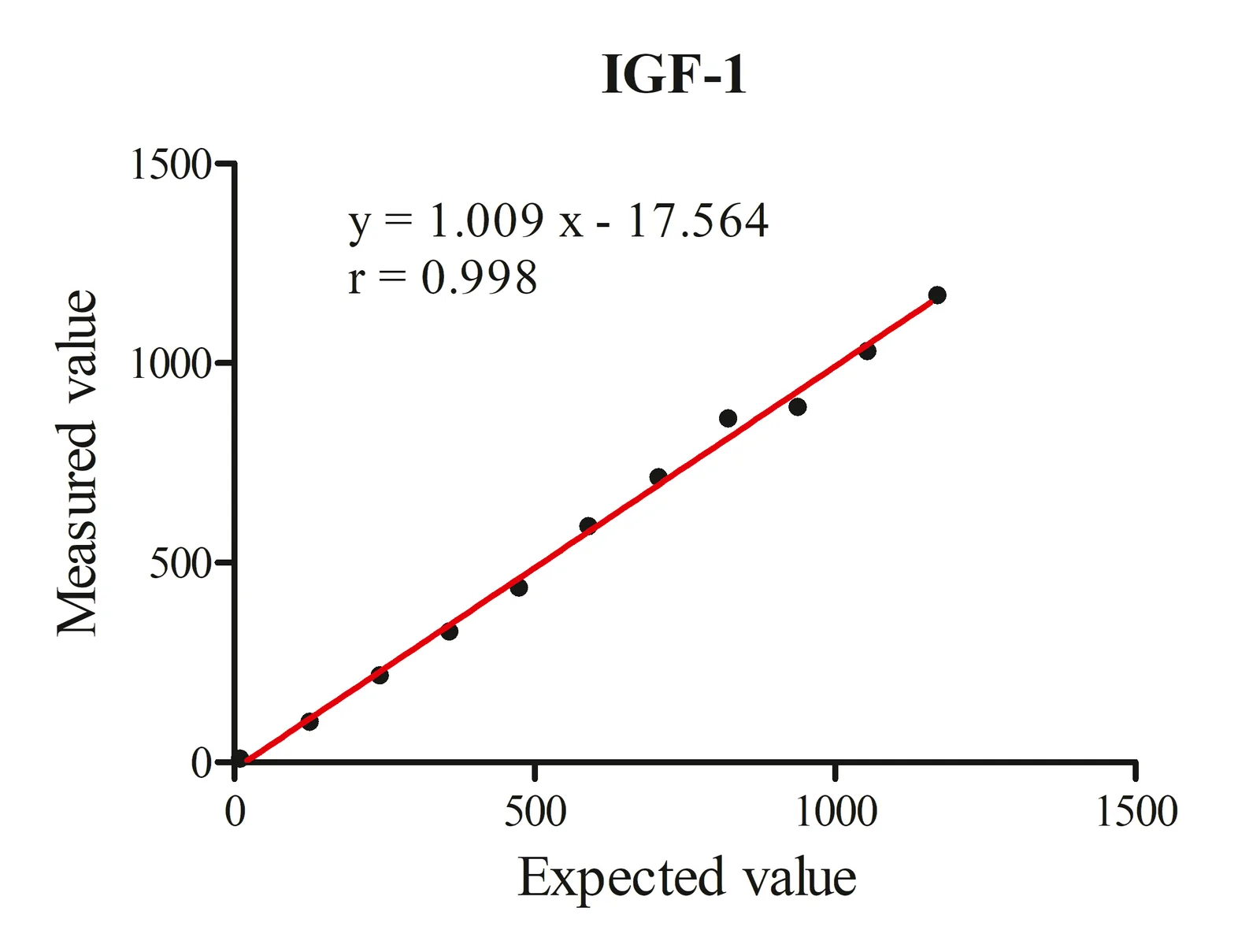
Linearity assessment by dilution for IGF-1. X-axis was expected value of IGF-1, and y-axis was measured value of IGF-1.

**Supplemental Fig. 2:**
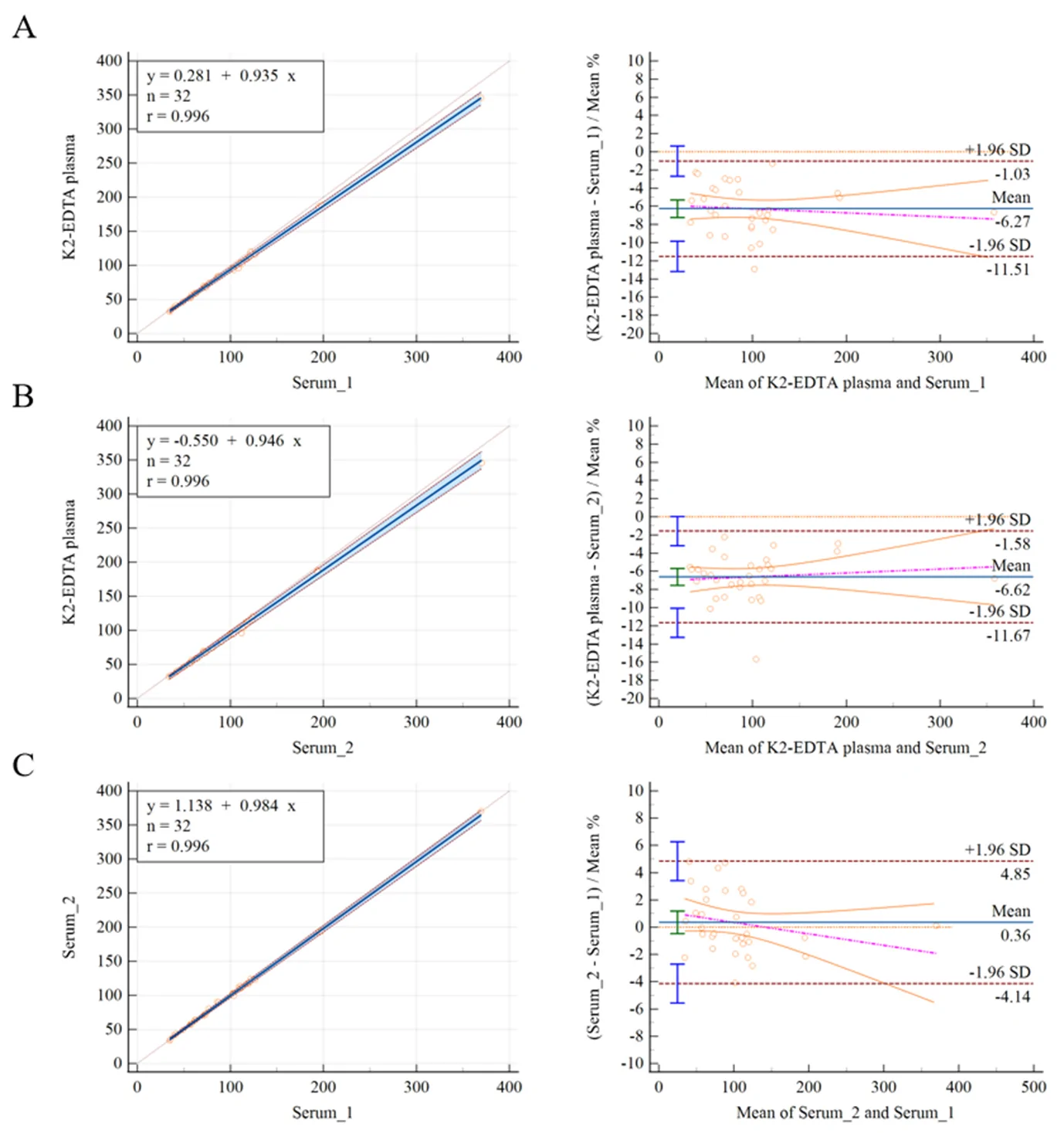
Passing-Bablok regression plot and Bland-Altman plot consistency test of serum and plasma.

## Data Availability

Data will be made available on request.

## Appendix A Supplementary data

Supplementary Table S1 Imprecision Performance Evaluation.

Supplementary Table S2 Spiked recovery test for accuracy evaluation.

Supplementary Table S3 Evaluation of lower limit of quantification performance.

Supplementary Table S4 Specificity Evaluation.

Supplementary Table S5 Results of sample storage stability tests.
